# Supplementary figures and images for: Biofilm-coated microbeads and the mouse ear skin: An innovative model for analysing anti-biofilm immune response in vivo
Source: PLoS One. 2020 Dec 4;15(12):e0243500. doi: 10.1371/journal.pone.0243500 (PMC7717515; doi:10.1371/journal.pone.0243500)

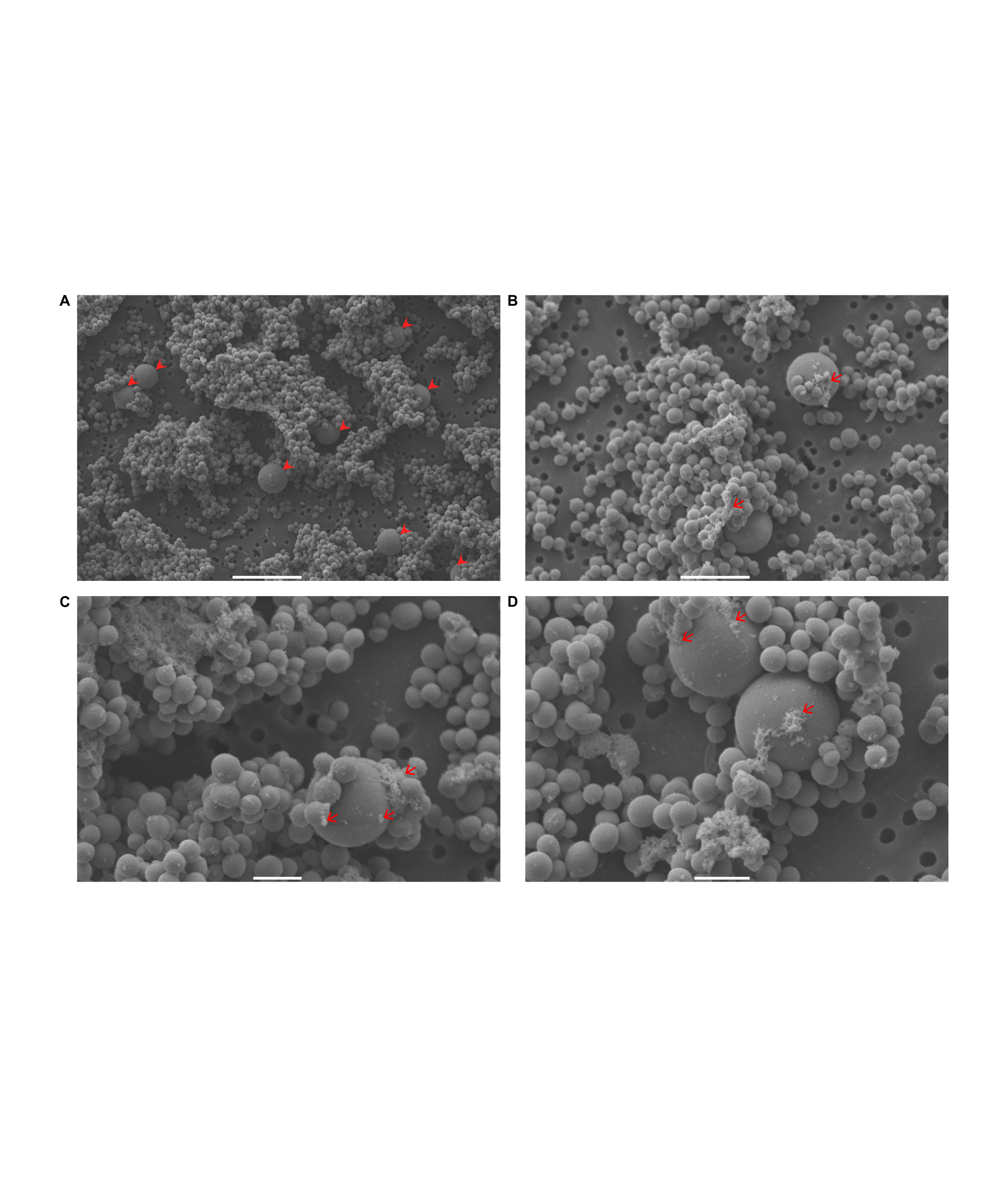

Supplement: S1 Fig — SEM micrographs of biofilm-coated microbeads at X2000 (A), X4000 (B), X7000 (C) and X8000 (D) magnification. Filled red arrowheads in (A) indicate microbeads and red arrows in (B), (C) and (D) indicate biofilm extracellular matrix. Scale bar: 10 μm (A), 5 μm (B), 2 μm (C), 2 μm (D). (TIF) [file pone.0243500.s001.tif]
